# Field measurements reveal exposure risk to microplastic ingestion by filter-feeding megafauna

**DOI:** 10.1038/s41467-022-33334-5

**Published:** 2022-11-01

**Authors:** S. R. Kahane-Rapport, M. F. Czapanskiy, J. A. Fahlbusch, A. S. Friedlaender, J. Calambokidis, E. L. Hazen, J. A. Goldbogen, M. S. Savoca

**Affiliations:** 1 California State University, Fullerton, Fullerton, CA USA; 2 Hopkins Marine Station, Stanford University, Pacific Grove, CA USA; 3 Cascadia Research Collective, Olympia, WA USA; 4 Institute for Marine Sciences, University of California, Santa Cruz, Santa Cruz, CA USA; 5 Environmental Research Division, NOAA Southwest Fisheries Science Center, Monterey, CA USA

**Keywords:** Conservation biology, Ecophysiology, Marine biology

## Abstract

Microparticles, such as microplastics and microfibers, are ubiquitous in marine food webs. Filter-feeding megafauna may be at extreme risk of exposure to microplastics, but neither the amount nor pathway of microplastic ingestion are well understood. Here, we combine depth-integrated microplastic data from the California Current Ecosystem with high-resolution foraging measurements from 191 tag deployments on blue, fin, and humpback whales to quantify plastic ingestion rates and routes of exposure. We find that baleen whales predominantly feed at depths of 50–250 m, coinciding with the highest measured microplastic concentrations in the pelagic ecosystem. Nearly all (99%) microplastic ingestion is predicted to occur via trophic transfer. We predict that fish-feeding whales are less exposed to microplastic ingestion than krill-feeding whales. Per day, a krill-obligate blue whale may ingest 10 million pieces of microplastic, while a fish-feeding humpback whale likely ingests 200,000 pieces of microplastic. For species struggling to recover from historical whaling alongside other anthropogenic pressures, our findings suggest that the cumulative impacts of multiple stressors require further attention.

## Abstract (web summary)

Combining microplastic data from the California Current Ecosystem with high-resolution foraging measurements from 191 tag deployments on blue, fin and humpback whales, this study quantifies plastic ingestion rates and routes of exposure risk in filter-feeding megafauna.

## Introduction

The Anthropocene Ocean is marked by the rapid proliferation of pollution, including noise^1^, chemical^2^, biological (e.g., biotoxins and pathogens)^3^, and plastic pollution^4^. Plastic production and disposal has risen more than twentyfold over the last half century and is projected to worsen through at least 2050^5,6^. Plastic consumption by wildlife, either directly by ingestion from the environment or indirectly via trophic transfer from prey, has become a pervasive phenomenon since plastic debris was first reported in marine food webs half a century ago^7,8^. At least 1500 species have been reported to ingest plastic^9^, particularly microparticles including microplastics (plastic pieces 0.001–5 mm) and microfibers (pieces 0.8–0.9 mm with a median diameter of 16.7 µm)^10^. However, the route of exposure, the extent, the effects, and the bioaccumulation of ingested plastic are understudied or unknown in most natural systems.

Determining exposure route and intensity are crucial first steps to projecting risk and mitigating harm for individuals and populations. Wildlife can confuse plastic for food but may also inadvertently ingest plastic that is adjacent to, attached to, or within food items. For top predators in particular, secondary ingestion of plastics via trophic transfer can be a dominant exposure route^11^. This ingested plastic may have deleterious effects on marine consumers; however, most of these threats are poorly understood^12^. While some taxa have been extensively studied for plastic ingestion, including marine fish^13^, sea turtles^14^, and seabirds^15^, this information is limited for marine mammals, particularly cetaceans. Baleen whales (Mysticeti) are perceived to be at high exposure risk of microplastic ingestion due to their diet, habitats, and filter-feeding behaviors^16–18^. Recent studies investigating stomach contents and fecal samples from baleen whales agree that plastic ingestion is ubiquitous, but disagree on the magnitude of plastic consumption^19,20^ (Table 1).

**Table 1:** Microplastic ingestion estimates or measures for similar species from other studies compared to the present study

Species	Region	Individuals studied	Measured or predicted plastic concentration in GI material (pp kg^−1^)	Estimated daily plastic ingestion (particles d^−1^)	Reference
*B. musculus* *B. physalus* *M. novaeangliae*	Eastern North Pacific	126 29 65	NA	5.30 × 10^6^−1.74 × 10^7^ 2.99–9.96 × 10^6^ 1.03–3.12 × 10^5^ (fish-feeding); 2.12–6.37 × 10^6^ (krill-feeding)	Present study*
*B. brydei, B. borealis*	Hauraki Gulf, New Zealand	21	5333 ± 4000	3.41 × 10^6^	Zantis et al., 2022^⊗^
*B. physalus*	North Atlantic	25	57	3.9–7.7 × 10^4^	Garcia-Garin et al. 2021†
*M. novaeangliae*	Eastern North Pacific	NA	2–6	6.0 × 10^4^−1.2×10^5^	Alava 2020^§^
*M. novaeangliae*	Eastern North Atlantic	1	NA	1.6 × 10^2^	Besseling et al. 2015^¶^
*M. novaeangliae*	Eastern North Pacific	NA	667	>3.0 × 10^5^	Desforges et al. 2015^#^
*B. physalus*	Mediterranean Sea	5 (tissue contaminants only)	NA	3.6 × 10^3^	Fossi et al. 2012**

Each study has specific caveats and conditions (presented in the caption, connected by unique superscript) that should be accounted for:

*We present the results of the medium scenario from the present study, in which we did not measure microplastics in whale scat.

^⊗^Value extrapolated from whale scat.

^†^Predicted daily rates from stomach contents.

^§^Extrapolations of bioaccumulation by the author, assuming a 30t whale.

^¶^Data from one stranded and emaciated individual.

^#^Did not estimate plastic ingested directly from seawater.

**Unable to estimate plastic ingested via trophic transfer. Source data are provided as a Source Data file.

The foraging behavior of filter-feeding megafauna including baleen whales is driven by the presence of dense but ephemeral patches of prey^21,22^. In many ecosystems, forage fish and zooplankton—the most common prey items of baleen whales—are regularly found to ingest microplastics, as the sizes of microplastics and the biological particles available for consumption are similar^23–27^. Therefore, trophic transfer has the potential to be the primary mechanism of ingestion. Although past research has suggested that filter-feeding megafauna, including baleen whales, are exposed to high quantities of microplastics specifically because they filter debris-laden water^17^, more recent work empirical has reported that baleen whales take in the majority of their microplastics from their prey^19^. Baleen whales have been found with microplastics in their gastrointestinal tract and fecal material^19,20,28^, and plastic-derived contaminants have been identified in their blubber^29^. However, the quantity of microplastics consumed by whales remains unknown. Without this information, it is difficult to develop exposure risk assessments to understand potential health effects or establish strategies to support mitigation for species- or ecosystem-based management plans.

Mysticetes use keratinized baleen plates to retain and ingest small fish and zooplankton. Among baleen whales, rorquals (family: Balaenopteridae) exhibit a two-step lunge feeding process, consisting of a high-speed engulfment event followed by a period of filtration through the baleen plates^30^. In the California Current Ecosystem (CCE), rorqual whales feed on krill swarms (primarily *Thysanoessa spinifera* and *Euphausia pacifica*) and forage fish schools (e.g., *Engraulis mordax* and *Clupea pallasii*)^31–33^.

While it remains untested how prey type affects the quantity of plastic ingested by most large predators, krill-feeding rorqual whales in the CCE filter fivefold more water and consume fourfold more prey than fish-feeding individuals^34^. Moreover, microplastic ingestion exposure risk, a product of engulfment capacity and feeding rates, may also be body size dependent. Previous research on rorqual whales has demonstrated strong effects of body size on engulfment capacity (i.e., volume engulfed during each feeding lunge) and feeding rate (i.e., number of lunges per dive)^35,36^. Engulfment capacity increases as rorqual whale body mass and length increase^35,37^. Conversely, feeding rates—which are influenced by body size^36^, prey type^38^, and predator-prey size ratios^39^ – generally decrease with increasing body size, similar to other physiological rates (e.g., heart rate and reproductive rate)^40,41^. No prior research has empirically evaluated plastic ingestion exposure risk across multiple baleen whale species and prey types. Clarifying the effect of body size and prey preference on microplastic ingestion will help establish which rorqual whales may face greater toxicological burdens from microplastic ingestion.

Chemical biomarkers and spatial overlap models have been developed to assess the likelihood and extent of ingestion and the associated risk to whale health^29,42^. However, prior work uses coarse metrics of foraging and spatially mismatched plastic concentrations to predict risk. For example, while the total overlap between microplastic pollution and fin whale (*Balaenoptera physalus*) habitat is extensive^17^, the majority of this overlap occurs at low latitudes where mating and birthing generally occurs^43^ and feeding is limited, though there are some notable exceptions (e.g., resident blue whales, *B. musculus*, in the low latitude Indian Ocean^44^). Other studies have generated ecotoxicological risk assessments while acknowledging a lack of field data to inform their models^18^. Moreover, most information on microplastic distributions comes from surveys of surface waters (top meter), and while rorquals occasionally forage at the surface^45^, they often feed at depth (50-300 m) where plastic distribution data is limited^22,46^.

In the CCE, there is baseline data on microplastic concentrations in the water column that overlaps with critical foraging habitat for humpback (*Megaptera novaeangliae*), fin, and blue whales. In this region, mean surface water concentrations of microplastics are ~1 particle m^−3^, but are severalfold higher near human population centers^47–49^. Measurements have found microplastic concentrations between 200 m and 600 m depths to be tenfold higher than those measured at the surface^50^. Furthermore, in the CCE, microplastics have been found in krill and forage fish^51,52^, both key mysticete prey species.

Here, we estimate microplastic ingestion by blue, fin, and humpback whales foraging on two prey types, krill and forage fish. We do so with a model built using high-resolution, spatially coherent field data on predator, prey, and plastic that can be adapted for other filter-feeders and can be updated as newer data becomes available. Mechanistically, we test the factors that drive exposure risk across species and individuals, including whether rorquals ingest more microplastics directly from the water column or indirectly as trophic transfer via their prey. We predict that krill-feeding whales will be at greater risk of exposure than fish-feeding whales due to higher foraging rates, absolute prey consumption, and the differences in microplastic contamination of prey^34^. Finally, we suggest that the opposing body-size dependent patterns of engulfment capacity and feeding rate will explain mass-specific rates of microplastic ingestion. Taken together, these results represent a first step towards clarifying the potential chemical and physiological effects of ingested microplastics on whales and other filter-feeding megafauna.

## Results and discussion

**Fig. 1:**
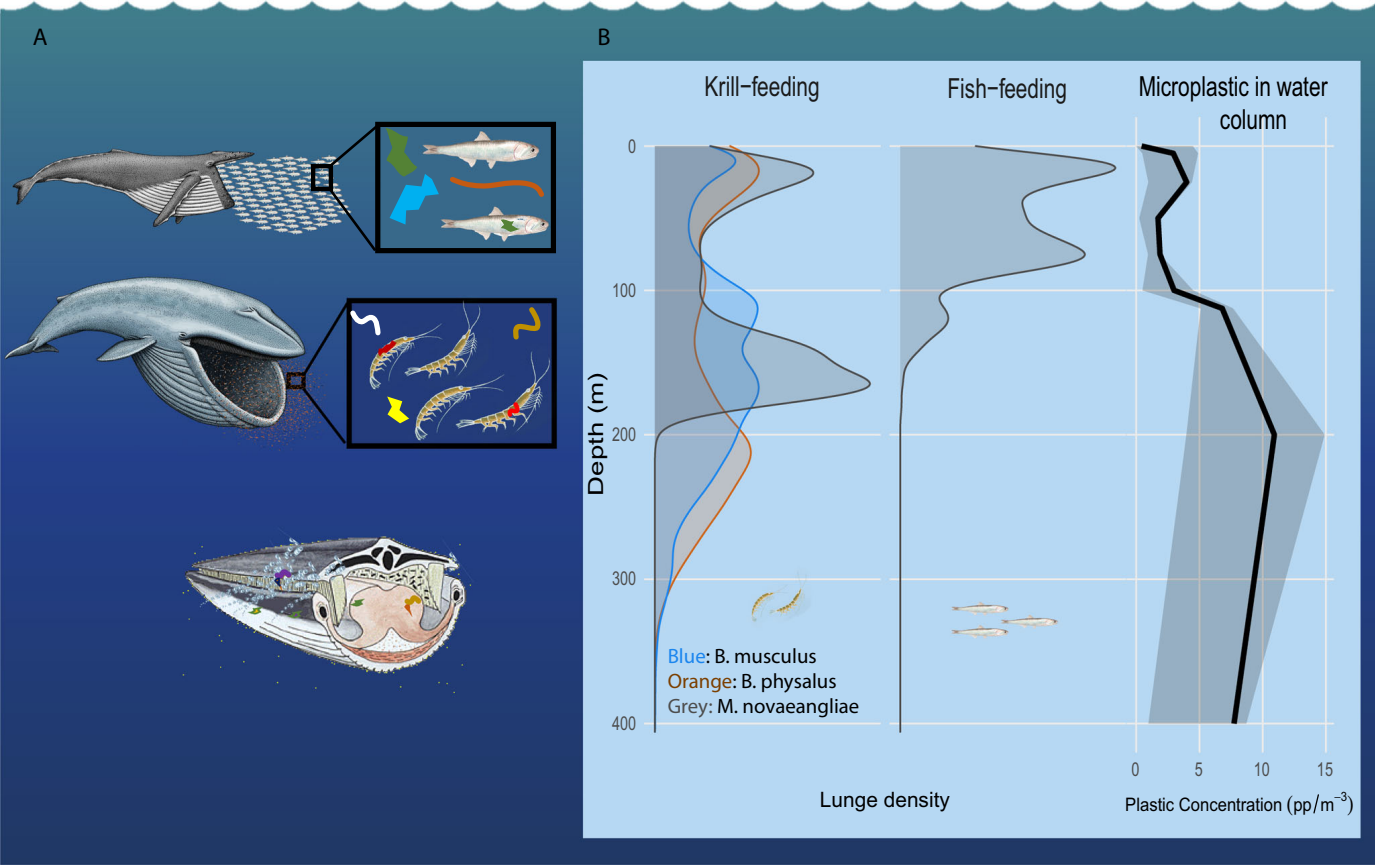
Depth of rorqual whale foraging in relation to microplastic in the water column. **a** Plastic ingested by whales day^−1^, modeled as the sum of (i) plastic filtered from water per day and (ii) plastic consumed in prey per day. We created three scenarios to capture the range of possible exposure risk of plastic ingestion, low, medium, and high, since some of the variables lack comprehensive data; **b** Lunge depths from deployments in Monterey Bay aligned with the depth profile of plastic concentration in Monterey Bay. Whales and prey items were illustrated by Alex Boersma and the cut-away filtration diagram was illustrated by Scott Landry at Center for Coastal Studies. Source data are provided as a Source Data file.

**Fig. 2:**
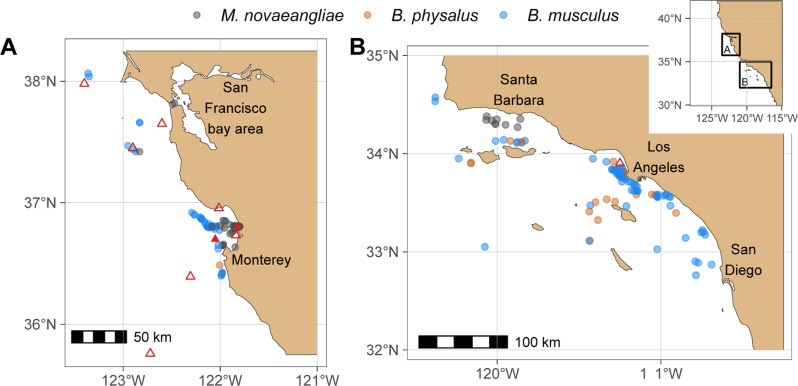
Locations of tag deployments, plastic sampling locations, and prey data collection used in this analysis. Filled red triangles represent plastic sampling locations at depth^50^ and hollow red triangles represent surface locations^47^. Circle color represents different rorqual species (see legend). **a** Northern California study sites; **b** Southern California study sites. Source data are provided as a Source Data file.

To estimate daily plastic consumption, we used fisheries acoustics to assess prey swarm density, airborne drones to estimate whale engulfment capacity, and high-resolution tag deployments of blue, fin, and humpback whales to quantify feeding rates. We merged these data with plastic ingestion rates by common rorqual whale prey species and depth-integrated distributions of plastic pollution in the CCE (Figs. 1, 2). We recorded 36,487 lunge-feeding events on 191 tag deployments and integrated 8 vertical water samples of microplastics.

**Fig. 3:**
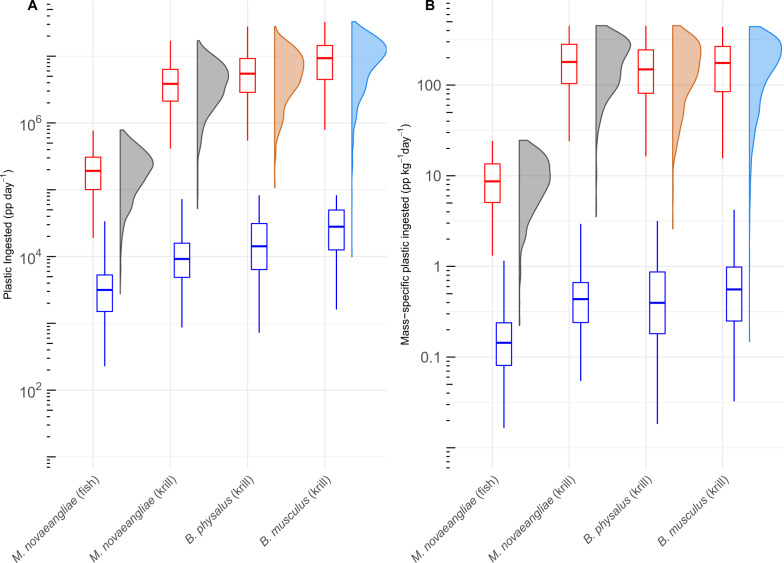
The results of the medium exposure risk scenario for *n* = 191 biologically independent animals, broken up by prey type and species. Red boxplots represent microplastics consumed through prey while blue boxplots represent microplastics retained by filtration. Data are presented as median values and interquartile range (25^th^−75^th^), note the logarithmic scale. The density plots represent the total microplastic ingested per feeding day (filtration + feeding). **A** Total number of plastic particles potentially ingested per day via filtration and prey consumption; **B** Mass-specific (whale mass, in kilograms) total plastic particles per day. Source data are provided as a Source Data file.

In the most likely scenario, krill-feeding rorqual whales are predicted to ingest 5.7 × 10^6^ (4.0 × 10^6^–1.1 × 10^7^) small synthetic particles during each day of intensive feeding (median, Q1–Q3; Fig. 3, Table 2). Over the course of a feeding season—conservatively assumed to encompass 90–120 days of heavy feeding in the region—a large blue whale may ingest over one billion pieces of microplastic during this period. With this improved empirical calculation of the number of microplastic pieces ingested, we can begin to estimate the ingested mass of microplastics which will help guide future work on the ecotoxicological burdens of ingested microplastics. The mass of individual microplastics is governed by factors including size, morphology, and chemical composition of the microplastic. As a result, the range of microplastic weights spans a twentyfold range, from 0.23 mg to 4 mg per microplastic piece^18,53^. Using this range, we estimate a blue whale would consume 2.51 to 43.6 kg of microplastics per day. Future research would be useful to increase the precision of these estimates and analyze the chemical composition of ingested microplastics to better assess ecotoxicological risk of microplastics to filter-feeding megafauna.

**Table 2:** The results of the medium exposure risk scenario, per species and prey type

Species	*B. physalus*	*B. musculus*	*M. novaeangliae* (fish feeders)	*M. novaeangliae* (krill feeders)
**Median Lunge Count (25th**–**75th** **Q1–Q3)**	177.21 (96.54–291.29)	190.77 (95.20–288.32)	99.66 (63.28–158.26)	285.45 (169.05–442.39)
**Median Plastic Pieces Retained from Water (25**^**th**^–**75th** **Q1–Q3)**	1.55 × 10^4^ (6.59 × 10^3^–3.69 × 10^4^)	3.59 × 10^4^ (1.54 × 10^4^–6.29 × 10^4^)	3.17 × 10^3^ (1.50 × 10^3^–5.30 × 10^3^)	9.20 × 10^3^ (4.86 × 10^3^–1.59 × 10^4^)
**Median Plastic Pieces Retained from Prey (25th**–**75**^**th**^ **Q1–Q3)**	5.70 × 10^6^ (2.99 × 10^6^–9.93 × 10^6^)	1.09 × 10^7^ (5.29 × 10^6^–1.73 × 10^7^)	1.01 × 10^5^ (3.08 × 10^5^–1.96 × 10^5^)	3.85 × 10^6^ (2.12 × 10^6^–6.36 × 10^6^)
**Median Total Plastic Pieces (25th**–**75th** **Q1–Q3)**	**5.73** **×** **10**^**6**^ **(2.99** **×** **10**^**6**^–**9.96** **×** **10**^**6**^**)**	**1.09** **×** **10**^**7**^ **(5.30** **×** **10**^**6**^–**1.74** **×** **10**^**7**^**)**	**1.96** **×** **10**^**5**^ **(1.03** **×** **10**^**5**^**−3.12** **×** **10**^**5**^**)**	**3.86** **×** **10**^**6**^ (**2.12** **×** **10**^**6**^–**6.37** **×** **10**^**6**^)
**Total Plastic Pieces Retained from Water / Animal Mass (kg) (25th**–**75th** **Q1–Q3)**	0.40 (0.18–0.87)	0.56 (0.25–0.98)	0.14 (0.08–0.24)	0.44(0.24-0.66)
**Total Plastic Pieces Retained from Prey / Animal Mass (kg) (25th**–**75th** **Q1–Q3)**	148.88 (81.37–244.19)	175.27 (84.74–267.33)	8.68 (5.06–13.51)	179.53 (104.02–281.89)
**Total Plastic Pieces / Animal Mass (kg) (25th**–**75th** **Q1–Q3)**	**149.20 (81.66**–**245.13)**	**175.62 (85.01**–**268.50)**	**8.81 (5.15-13.75)**	**180.25 (104.29**–**282.39)**

Each row contains the median and interquartile range for the modeled result. Source data are provided as a Source Data file.

Bolded rows are those that combine the plastic ingested from both water and prey.

Our high-risk scenario represents the threat foraging rorqual whales may face in areas close to human population centers or may face if plastic production and disposal continues at its current pace. Based on the high-risk scenario, blue whales are projected to ingest 4.62 × 10^7^−1.52 × 10^8^ microplastic pieces per day (Q1–Q3; Supplementary Table 2). In contrast, our low-risk scenario represents areas of lesser concern, or a future where plastic influx to the marine environment is ameliorated. Under the low-risk scenario, blue whales are projected to ingest 9.66 × 10^4^–3.13 × 10^5^ pieces per day (Q1–Q3; Supplementary Table 3).

Fish-feeding humpback whales are projected to ingest an order of magnitude less microplastics than krill-feeding individuals of the same species. We estimate that a humpback whale consuming anchovies in the CCE will ingest 1.03–3.12 × 10^5^ (Q1–Q3) small synthetic particles, as compared to 2.12–6.37 × 10^6^ (Q1–Q3) for a krill-feeding humpback, during each feeding day. Fish-feeding rorquals are projected to ingest 98.5% of their total plastic load through prey, while krill-feeding rorquals likely ingest >99% of all plastic via their prey. As shown in other studies^34,38^, we find that lunge-feeding rates for fish-feeding individuals are threefold lower than for krill-feeding individuals (63–158 vs. 169–442 lunges d^−1^, respectively) (MCMCglmm, *p* < 0.0001), which may lead to these large differences. In addition, we assumed that fish with a positive incidence of microplastic ingestion contain only one piece of microplastic per fish, which is likely an underestimate. These factors combined may lead to underestimates of microplastic ingestion by fish-feeding mysticetes.

Most of the feeding across all species (99.6%) took place below the surface (>1 m depth) and feeding often occurs well beneath the surface. For example, 83.75% blue whale lunges occur deeper than 50 m (Fig. 1, Supplementary Table 4). This finding—along with differences between plastic concentrations in surface waters and at depth^50,54^ (Fig. 1)—suggests that using surface concentrations of microplastics to generate quantitative risk assessments for predators that feed at depth may be inappropriate. Krill-feeding humpbacks tend to forage deeper in the water column (69.05% of lunges occurring deeper than 50 m, Fig. 1, Supplementary Table 4) than fish-feeding humpbacks (51.80% of lunges occurring deeper than 50 m, Fig. 1, Supplementary Table 4). Deeper foraging may lead to higher plastic ingestion from filtered water as the greatest concentration of microplastic in Monterey Bay was found between 200–300 m^50^. However, for all individuals, the vast majority of plastic consumed occurred through secondary consumption of prey that had previously ingested and not yet egested microplastics.

The finding that rorqual whales may ingest several million microplastics per day suggests that filter-feeding megafauna may be at risk via the cumulative physiological and toxicological burdens from this debris. A considerable majority of all microplastic ingested is likely a result of trophic transfer from prey (>98% for all species and prey types considered), and less so from the retention of microplastic suspended in seawater. While mysticetes were already perceived to be at high risk for microplastic ingestion^17^, our projections—derived from empirical field data on plastic concentrations, prey consumption, and rorqual foraging—were higher than previously hypothesized (Table 1). This is because rorqual whale prey consumption and water filtered are both higher than expected^34^, a result that stems from the positive allometry of the engulfment apparatus^35^.

Previous studies presented first estimates of daily microplastic ingestion that ranged between 10^2^–10^6^ pieces per day (Table 1). Microplastic ingestion rates in those studies span four orders of magnitude. Our study, the only one to analyze whale foraging behavior as well as depth-resolved microplastic concentrations, suggests that the highest values within this range are most likely to be accurate, and even those may underestimate microplastic consumption. We did not include the elimination rate of microplastics by rorqual whales in our analysis as no empirical data exists. Laboratory studies on fish suggest that nearly all ingested microplastics are eliminated through defecation in ~7 days^55–57^. However, as microplastics get smaller—the nanoparticle range (particles ≤ 0.001 mm^58^)—their likelihood of incorporation and bioaccumulation in body tissues increases^59^. There is mounting evidence that particles <100 µm can translocate into and through the intestinal wall, become lodged in body tissues, and lead to physical impacts at the cellular level^60–62^. Determining the elimination (via defecation) and incorporation rate of nanoplastics is critical to understanding their long-term effects.

While nanoplastics are abundant in marine environments, mechanical and chemical digestion of plastic-consuming wildlife further exacerbates this issue. For example, Antarctic krill (*E. superba*) can fragment microplastics into nanoplastics during their digestive processes^63^; North Pacific krill species may have the same ability, and whales themselves may fragment ingested microplastics into nanoparticles through digestive action. Although we cannot empirically quantify bioaccumulation of microplastics, we can consider extreme levels of microplastic ingestion a leading indicator of nanoplastic ingestion by whales. This emerging issue needs to be closely monitored and reassessed as more data becomes available.

**Fig. 4:**
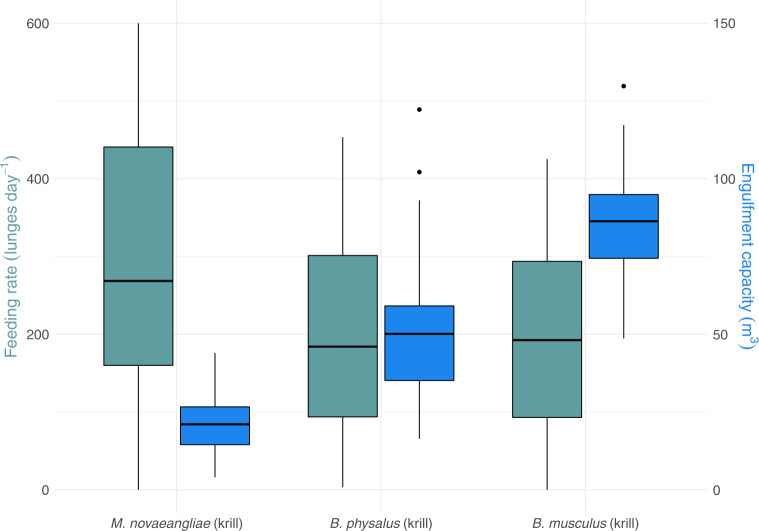
Engulfment capacity (m^3^) and feeding rate (lunges day ^−1^) display opposite trends when compared to body size for *n* = 191 biologically independent krill-feeding animals. Body size increases from *M. novaeangliae* to *B. musculus*. Engulfment capacity increases and feeding rate decreases with body size. Microplastic ingestion exposure risk, a product of engulfment capacity and feeding rates, is likely also body-size dependent. Data are presented as median values and interquartile range (25th−75th). Source data are provided as a Source Data file.

Mass-specific microplastic ingestion was similar across krill-feeding species (Fig. 3B). We expected blue whales to be at a disproportionately greater exposure risk from microplastic ingestion because of their proportionally larger engulfment capacities compared to fin and humpback whales. However, because of their lower feeding rates, across days and throughout the foraging season, they may encounter fewer pieces of microplastic in both the water and prey (krill) (Fig. 4). Because of the competing effects between foraging rate and engulfment capacity, microplastic exposure risk may not be tied to body size, but instead may be influenced by the frequency of microplastic occurrence in prey^13,51,64^ and the distribution of microplastic pollution in the water column^50^.

Our simulations included a conservative approach towards incorporating plastic frequency of occurrence (FO), assuming that individual prey items that had ingested plastic contained only one microplastic particle. However, many prey species that ingest plastic, particularly fish, may be found to contain numerous pieces of ingested plastics^64–67^, and can themselves consume prey that has ingested microplastic. Additionally, the size and form of plastic consumed by krill and fish may be different^51,63,65^ and lead to disparate effects. Future work should incorporate these factors to generate a more accurate estimate of plastic ingestion and its effects.

In the CCE, plastic ingestion data is limited for species at the base of marine food webs (e.g., zooplankton and forage fish). While some plastic ingestion data from these primary and secondary consumers in this region exists^50–52^, sample sizes are limited and long-term monitoring is lacking. In addition, the plastic FO values in prey we used for our most likely scenario were retrieved from decade-old field data^51,52^ and may have increased over time^13^. Recent work in Monterey Bay suggests that the values used in the high-risk scenario may in fact be more likely^68^. As such, we used plastic FOs in congeneric prey species from the Northwest Pacific—specifically the Yellow, East, and South China Seas^24,25,69^—as a potential future scenario for the CCE (i.e., our high-risk scenario) where plastic pollution continues to rise in the coming decades, as is predicted^5^. Collecting ongoing data from ecologically critical prey species is essential as microplastic rates continue to increase. Doing so will allow for a more complete accounting of plastic incursion into marine food webs that lead to apex predators including predatory fish, seabirds, and marine mammals.

While some mysticete populations and lineages surface lunge or skim-feed^45,70^, this foraging strategy is the exception rather than the rule. Moreover, gray whales and some populations of humpback whales suction feed in the sediment to extract invertebrates^71^. As the seafloor is a primary sink for synthetic marine debris^72,73^, there may be considerable risk to gray and humpback whales feeding in the substrate. An analysis of synthetic debris in both sediment and prey species across foraging habitats has yet to be conducted. Beyond the North Pacific, Eden’s whales (*B. edeni*) foraging in heavily polluted Southeast Asian seas are likely strong indicators for microplastic ingestion^74^. In addition, the Mediterranean Sea and the Gulf of Mexico are two semi-enclosed basins of concern. These regions are known for high concentrations of plastic debris and other contaminants, face extreme and encroaching anthropogenic pressures, and are home to two endemic mysticetes—a genetically distinct fin whale population in the Mediterranean^75^ and the newly described, and critically endangered, Rice’s whale (*B. ricei*) in the Gulf of Mexico^76^.

Ram-feeding megafauna, such as balaenid whales, filter approximately fourfold more water on a daily basis than a similarly sized rorqual^34^, but their plastic ingestion has never been estimated or measured. Two balaenid species—the North Pacific (*Eubalaena japonica*) and North Atlantic (*E. glacialis*) right whales—are also critically endangered, and the exposure and effects of chronic pollutants in these species is almost entirely unknown. More research is needed to understand the risk of plastic ingestion to other ram-feeding megafauna including mobulid rays, and basking (*Cetorhinus maximus*) and megamouth sharks (*Megachasma pelagios*). Whale shark (*Rhincodon typus*) fecal samples have recently been shown to contain microplastics (mean: 2.8 microplastics g^−1^ feces)^77^. This, along with previous work that detected microplastic-derived contaminants in whale shark skin^78^, indicates that microplastic ingestion can be tracked using numerous techniques that when analyzed holistically, can clarify both the rate and the long-term effects of ingestion. A concerning but unstudied possibility is that plastic ingestion by prey may reduce the energetic quality of these food items. For capital breeders like mysticetes, even small reductions in the prey quality can have important consequences on reproduction and fitness^68^. Studying these consequences will strengthen the use of baleen whales as indicators of pollution and habitat change in their ecosystems^53^.

While the influx of plastic pollution into marine food webs is of increasing concern, the mechanism(s) and extent of plastic ingestion for many marine predators is unknown. Here we show that rorqual whales—the largest consumers on earth—are also likely to be the largest consumers of plastic on an absolute basis and that the majority of their plastic ingestion likely originates through trophic transfer. Baleen whales ingesting and egesting large quantities of microplastic on their feeding grounds may affect the vertical or horizontal distribution of plastic in still-unknown ways. Our findings provide a quantitative estimate of microplastic consumption that can be tested with additional field data and lay the foundations for clarifying microplastic bioaccumulation in marine food webs. Linking microplastic ingestion rates to the effects of microplastic on marine biota is a crucial next step to meet the conservation and sustainability challenges of the Anthropocene.

## Methods

### Tag deployment and data processing

We used Customized Animal Tracking Solutions (CATS) tag, Digital Acoustic Recording (DTAG) tag, and medium duration dart-tag deployments in our analysis. 29 fin whale, 126 blue whale, and 65 humpback whale deployments were collected between 2010–2019 and recorded whales foraging within the CCE, specifically within the Monterey Bay, Channel Islands, Gulf of the Farallones, and Cordell Bank National Marine Sanctuaries (Fig. 2, Supplementary Table 1). Some of these tag data have been published^79–82^. All fieldwork was conducted under National Marine Fisheries Service permits 16111, 14809, 19116, 21678, 20430, and National Marine Sanctuary permits MULTI-2017-007 and MULTI-2019-009, in accordance with Stanford University IACUC (#30123).

For all CATS tag deployments, accelerometers (dynamic range ± 39.2 m s^−2^) were sampled at 400 Hz, magnetometers and gyroscopes (dynamic range 1000 deg s^−1^) were sampled at 50 Hz, and pressure was sampled at 10 Hz^83^. DTAGS were equipped with a pressure transducer (50 Hz), tri-axial accelerometers (250 Hz), magnetometers (50 Hz), and audio. Two styles of medium-duration (multi-day) dart tags were used in this study: Wildlife Computers TDR10-F (https://wildlifecomputers.com/our-tags/tdr/tdr10/) and Acousonde acoustic (http://www.acousonde.com) tags^82^. These tags contained a satellite transmitter and were attached with 3–4 stainless steel darts 4–6 cm long. Both tag types sampled depth, 3D accelerometry (≥32 Hz) and fast-acquisition GPS.

All data were decimated to 10 Hz before further analysis^84^. Tag orientation was corrected to whale-frame (the natural axes of the animal) using periods of known orientation, and animal orientation (pitch, roll and heading) was calculated in MATLAB (version 2014b)^83–85^. Continuous animal speed was determined using the amplitude of the tag vibrations^86^. Video and sound were recorded concurrently and were aligned with sensor data in MATLAB^84^. Rorqual lunges have a distinct kinematic signature. A lunge is confirmed by (1) fluking associated with a distinct speed maximum and (2) rapid deceleration with continued forward momentum, owing to the engulfed water mass^30^. We used this confirmation procedure to manually identify lunge-feeding events from the tag data^83^.

### Quantifying to microplastic ingestion exposure

To quantify microplastic ingestion exposure by foraging rorqual whales, we used:

1 $$P={Vr}\mathop{\sum}\limits_{d}{f}_{d}{C}_{d}+\left(\frac{{Vf}\,{\rho }_{y}}{{m}_{y}}{C}_{y}\right)$$

[Equation 1] Mathematical representation of model quantifying microplastic ingestion exposure

Plastic ingested by whales (*P*, in plastic pieces (pp) day^−1^) was modeled as the sum of (i) plastic filtered from water per day, accounting for depth, and (ii) plastic consumed in prey per day (Eq. 1). Since some of the variables are unknown or lack comprehensive data, we created three scenarios to capture the range of possible exposure risk of plastic ingestion: low, medium, and high. Each scenario simulation was run 1000 times (Fig. 3).

Microplastic filtered from water per day, accounting for depth, was modeled as the product of the volume of seawater engulfed per lunge (*V*, m^3^), the percentage of microplastic retained by baleen (*r*, unitless), the feeding events (*f*) per day within a known depth bin (*f*_d_, day^−1^), and the microplastic concentration within a known depth bin (*C*_*d*_, pp m^−3^). Microplastic consumed in prey was estimated as the product of: the prey density per feeding event (*ρ*_*y*_, kg m^−3^), the mass of an individual prey item (*m*_*y*_, kg ind^−1^), and the microplastic concentration in prey (*C*_y_, pp ind^−1^). The predicted distribution of microplastic ingestion, a product of a distribution of body sizes, feeding events, and prey biomass consumed, was estimated by applying a Monte Carlo simulation using package MCMCglmm in^87^ RStudio (v 4.0.3).

Feeding events per day within a known depth bin were estimated from sub-daily and daily tag data. Each deployment had a latitude and longitude associated with it (Fig. 2). Each lunge was associated with a depth, date, and time of day. The lunge depths were grouped into five depth bins: surface (0–0.5 m), sub-surface (0.5–5 m), shallow (5–50 m), moderate (50−150 m), and deep (>150 m). Date, latitude, and longitude were used to determine the sun angle and therefore how many hours of daylight, night, and twilight were within each deployment. Time of day and sun angle were used to group lunges into three diel periods, day, twilight, and night, thus creating a lunge rate per diel period for each species. Then the model combined a day, twilight, and night rate for each simulated whale, creating a distribution of daily lunge rates per species.

Volume of seawater engulfed per lunge (*V*, m^3^) was estimated using morphological data. We used published allometric equations to estimate the volume of engulfed water based on total body length^35^. We used drone images of 68 humpback, 178 fin, and 40 blue whales taken off the coast of California and historical records of whale length from California whale harvests^33^ to create a distribution of total body length.

Microplastic concentration in seawater within a known depth bin (*C*_*d*_, pp m^−3^) was estimated using two published distributions^47,50^ (Fig. 2). The distribution from ref. 50 extended from 5–1000 m. The distribution from ref. 47 provided data for the surface to 0.5 m. The microplastic distribution data was sorted into depth bins to match the lunges.

The percentage of plastic particles retained by baleen (*r*) is unknown. Preliminary tests were conducted by recording the microplastic retention of two pieces of blue whale baleen in a recirculating flow tank system. We pushed water carrying neutrally buoyant microplastic spheres of various sizes (5 mm, 2 mm, and 1 mm) into the baleen at a consistent speed. Video analysis indicated that baleen could retain plastic particles that fall within the microplastic designated size range (0.001–5 mm). We concluded that testing three percentages of plastic particle retention, 25%, 50%, and 75%, would provide a reasonable estimate for this unknown variable (A. Werth, pers. comm.).

Prey biomass density (*ρ*_*y*_, kg m^−3^) was calculated for both krill and anchovy, the two common prey items of rorqual whales foraging in Monterey Bay, according to the methods in refs. 34,38,79,80.

Little is known about plastic concentration in prey (*C*_y_, pp ind^−1^), specifically krill and anchovy, in Monterey Bay (but see ref. 68). However, there are previously published distributions of microplastic pieces within krill and anchovy for other locations and systems. Therefore, we set a low, medium, and high frequency of occurrence for both krill and anchovy based on conservative literature values^20,24,25,51,52,88^. We report the frequency of occurrence of microplastics, which is the number of individuals with plastic in their diet divided by the total number of individuals collected.

We set the low estimate to be a 0.01 frequency of occurrence (FO) in krill. This was the approximate FO back-calculated from gut content analyses of fin whales feeding in the remote North Atlantic^20^. The medium estimate (our ‘most likely’ scenario) of a 0.06 FO was found by^51^ in Northeast Pacific zooplankton (*Neocalanus cristatus* and *Euphausia pacifica*). We used a 0.50 FO for the high scenario, based on the work of ref. 69 in the South China Sea. This value is much greater than what was found by ref. 51 but less than that found by ref. 68. Although the ref. 68 study was conducted in Monterey Bay, the results were based on a limited sample size (five individual krill) and would have made our values even higher. We choose to use older studies with lower FOs that were based on greater sample sizes, recognizing that this may underestimate microplastic consumption. Monterey Bay’s proximity to urban centers and tenfold increase in plastic concentrations in surface seawater in the region from 2006–2007 to 2017–2019^47,49,89^ suggests that the krill plastic FO from ref. 69 may be possible in Monterey Bay krill in the near future. We assumed that each krill that contained plastic only contained one piece.

For anchovy, our low estimate was a 0.02 FO, based on the findings of ref. 88 in Pacific herring (*Clupea pallasii*) from coastal British Columbia. Fish-feeding humpbacks in the CCE eat primarily sardine and anchovy, but humpbacks specialize on herring in other parts of their range^90^. We used 0.30 FO for the medium scenario based on the findings of ref. 52 in Pacific anchovy (*Engraulis mordax*) collected in California. Finally, we used a high estimate of 0.77 FO, based on the findings of ref. 25. Although^25^ sampled Japanese anchovy (*Engraulis japonicus*) from Tokyo Bay, their results represent a probable frequency of occurrence in the future, especially in coastal waters near highly populated areas and with continuing production of microplastics. Some studies found an average of 0.13 and 0.11 microplastic pieces per individual fish, respectively^66,67^. However, others^64^ found that the average number of microplastic pieces found per fish was 1.8 and^68^ found an even greater average of seven microplastics per anchovy. We assumed that each individual fish that contained plastic only contained one piece, again recognizing this is likely an underestimate.

### Statistical analyses

We used generalized linear mixed models (GLMM) with parameters estimated via Markov Chain Monte Carlo (MCMC) algorithm to generate posterior probability distributions of our parameters of interest. These models were fit with the MCMCglmm package in R (v 4.0.5, https://www.r-project.org/)^87^. We ran our MCMC-GLMMs for 10,000 iterations with a 1000-run burn-in. We tested how humpback whale lunge rates may differ because of prey differences using a MCMCglmm.

### Reporting summary

Further information on research design is available in the Nature Research Reporting Summary linked to this article.

## Supplementary information

Supplementary Information

Reporting Summary

## Data Availability

Depth-integrated microplastic data can be found in refs. 47,50. Prey biomass and density data can be found in refs. 34,38,79,80. The risk of ingestion data generated in this study have been deposited in a GitHub repository at https://github.com/shirelkr/risk-of-microplastic-ingestion-by-filter-feeding-megafauna. Source data are provided with this paper.

## Source data

Source Data

## Code availability

Code for tag data analyses can be found in ref. 83. The code used in this study has been deposited in a GitHub repository at https://github.com/shirelkr/risk-of-microplastic-ingestion-by-filter-feeding-megafauna.
