# Supplementary material for: Field measurements reveal exposure risk to microplastic ingestion by filter-feeding megafauna
Source: Nat Commun. 2022 Nov 1;13:6327. doi: 10.1038/s41467-022-33334-5 (PMC9626449; doi:10.1038/s41467-022-33334-5)
Supplement: Supplementary file 1 — Supplementary Information [file 41467_2022_33334_MOESM1_ESM.pdf]

Title: Supplementary Information for ‘Field measurements reveal exposure risk to microplastic ingestion by filter-feeding megafauna’

Supplementary Table 1. Summary of rorquals species tagged, deployment duration, tag type, along with prey type. Source data are provided as a Source Data file.

**Species**	**Prey type**	**Tag type**	**Num. deployments**	**Tag time (hours)**
B. physalus	Krill	CATS	3	2.83 (0.74 – 4.16)
B. musculus	Krill	CATS	45	12.86 (0.77 – 43.38)
M. novaeangliae	Fish	CATS	34	6.56 (0.48 – 31.00)
M. novaeangliae	Krill	CATS	13	7.33 (2.11 – 12.18)
M. novaeangliae	Fish	Medterm	2	10.25 (2.37 – 18.12)
M. novaeangliae	Krill	Medterm	1	23.53 (23.53 – 23.53)
B. musculus	Krill	Medterm	14	204.35 (66.68 – 771.55)
B. physalus	Krill	Medterm	6	75.72 (20.40 – 120.93)
B. physalus	Krill	DTAG	20	5.14 (0.70 – 19.52)
B. musculus	Krill	DTAG	53	5.87 (0.51 – 34.37)

Supplementary Table 2. The results of the high-risk scenario, per species and prey type. Each row contains the median and interquartile range for the modeled result. Source data are provided as a Source Data file.

Species	B. physalus	B. musculus	M. novaeangliae (fish feeders)	M. novaeangliae (krill feeders)
**Median Lunge Count (25th – 75th IQR)**	185.49 (89.60-303.36)	200.05 (93.22- 308.69)	108.62 (62.79- 158.46)	260.63 (152.85- 449.46)
**Median Plastic Pieces Retained from Water (25th – 75th IQR)**	$2.34x10^4$ $9.84x10^3$- $5.06x10^4$	$5.76x10^4$ ($2.34x10^4$ – $1.01x10^5$)	$4.77x10^3$ ($2.31x10^3$- $7.84x10^3$)	$1.20x10^4$ ($5.96x10^3$- $2.20x10^4$)
**Median Plastic Pieces Retained from Prey (25th – 75th IQR)**	$5.02x10^7$ ($2.33x10^7$- $8.81x10^7$)	$9.83x10^7$ ($4.62x10^7$- $1.52x10^8$)	$5.03x10^5$ ($2.59x10^5$- $7.91x10^5$)	$3.04x10^7$ ($1.52x10^7$- $4.87x10^7$)
**Median Total Plastic Pieces (25th – 75th IQR)**	**$5.02x10^7$ ($2.33x10^7$- $8.82x10^7$)**	**$9.84x10^7$ ($4.62x10^7$- $1.52x10^8$)**	**$5.09x10^5$ ($2.62x10^5$- $8.00x10^5$)**	**$3.04x10^7$ ($1.52x10^7$- $4.88x10^7$)**
**Total Plastic Pieces Retained from Water / Animal Mass (kg) (25th – 75th IQR)**	0.61 (0.27- 1.27)	0.91 (0.36- 1.51)	0.22 (0.12- 0.36)	0.55 (0.31-0.95)
**Total Plastic Pieces Retained from Prey / Animal Mass (kg) (25th – 75th IQR)**	1317.13 (632.09- 2159.29)	1518.55 (713.60- 2355.65)	23.49 (13.44- 34.27)	1449.16 (774.24- 2304.37)
**Total Plastic Pieces / Animal Mass (kg) (25th – 75th IQR)**	**1317.97 (632.51- 2161.18)**	**1519.70 (713.87- 2357.35)**	**23.77 (13.64- 34.61)**	**1449.51 (774.54- 2305.66)**

Footnote: Bolded rows are those that combine the plastic ingested from both water and prey.

Supplementary Table 3. The results of the low-risk scenario, per species and prey type. Each row contains the median and interquartile range for the modeled result. Source data are provided as a Source Data file.

Species	B. physalus	B. musculus	M. novaeangliae (fish feeders)	M. novaeangliae (krill feeders)
**Median Lunge Count (25th – 75th IQR)**	193.37 (95.97-305.11)	188.09 (89.31- 278.33)	103.83 (59.23- 161.58)	256.18 (153.95- 427.78)
**Median Plastic Pieces Retained from Water (25th – 75th IQR)**	7793.55 (3402.24 - 16651.93)	18166.10 (7484.87-32463.51)	1583.78 (710.76-2803.72)	4313.48 (2214.55-7469.13)
**Median Plastic Pieces Retained from Prey (25th – 75th IQR)**	$1.03x10^5$ ($4.78x10^4$-$1.73x10^5$)	$1.82x10^5$ ($8.80x10^4$-$2.80x10^5$)	$1.24x10^4$ ($6.41x10^3$-$2.07x10^4$)	$5.87x10^4$ ($3.02x10^4$-$9.91x10^4$)
**Median Total Plastic Pieces (25th – 75th IQR)**	**$1.13x10^5$ ($5.24 x10^4$-$1.89x10^5$)**	**$2.02x10^5$ ($9.66 x10^4$-$3.13x10^5$)**	**$1.42x10^4$ ($7.19x10^3$-$2.36x10^4$)**	**$6.28x10^4$ ($3.25x10^4$-$1.09x10^5$)**
**Total Plastic Pieces Retained from Water / Animal Mass (kg) (25th – 75th IQR)**	0.2 (0.09- 0.42)	0.29 (0.12-0.49)	0.07 (0.04-0.12)	0.2 (0.11-0.32)
**Total Plastic Pieces Retained from Prey / Animal Mass (kg)**	2.65 (1.31-4.27)	2.84 (1.39-4.31)	0.57 (0.34-0.93)	2.66 (1.60-4.53)

**(25th – 75th IQR)**				
**Total Plastic Pieces / Animal Mass (kg) (25th – 75th IQR)**	**2.86 (1.46-4.73)**	**3.21 (1.54-4.85)**	**0.66 (0.38-1.05)**	**2.91 (1.71-4.91)**

Footnote: Bolded rows are those that combine the plastic ingested from both water and prey.

Supplementary Table 4. Lunge counts divided by depth bin, species, and prey type. Source data are provided as a Source Data file.

**Depth Bin**	**Species**			
	B. physalus, krill (count)	*B. musculus*, krill (count)	*M. novaeangliae*, krill (count)	*M. novaeangliae*, fish (count)
**Surface (0-0.5m)**	27	74	12	30
**Sub-Surface (0.5-5m)**	378	707	24	82
**Shallow (5-50m)**	1267	3495	1438	1548
**Moderate (50-150m)**	1331	9476	1756	1766
**Deep (>150m)**	3070	12559	1532	18
