# Supplementary material for: Field measurements reveal exposure risk to microplastic ingestion by filter-feeding megafauna
Source: Nat Commun. 2022 Nov 1;13:6327. doi: 10.1038/s41467-022-33334-5 (PMC9626449; doi:10.1038/s41467-022-33334-5)
Supplement: Supplementary file 2 — Reporting Summary [file 41467_2022_33334_MOESM2_ESM.pdf]

nature portfolio

Corresponding author(s): Shirel R. Kahane-Rapport

Last updated by author(s): 8/12/2022

Reporting Summary

Nature Portfolio wishes to improve the reproducibility of the work that we publish. This form provides structure for consistency and transparency in reporting. For further information on Nature Portfolio policies, see our Editorial Policies and the Editorial Policy Checklist.

Statistics

For all statistical analyses, confirm that the following items are present in the figure legend, table legend, main text, or Methods section.

n/a	Confirmed	
☐	☒	The exact sample size (n) for each experimental group/condition, given as a discrete number and unit of measurement
☐	☒	A statement on whether measurements were taken from distinct samples or whether the same sample was measured repeatedly
☒	☐	The statistical test(s) used AND whether they are one- or two-sided<br>*Only common tests should be described solely by name; describe more complex techniques in the Methods section.*
☒	☐	A description of all covariates tested
☐	☒	A description of any assumptions or corrections, such as tests of normality and adjustment for multiple comparisons
☐	☒	A full description of the statistical parameters including central tendency (e.g. means) or other basic estimates (e.g. regression coefficient) AND variation (e.g. standard deviation) or associated estimates of uncertainty (e.g. confidence intervals)
☒	☐	For null hypothesis testing, the test statistic (e.g. F, t, r) with confidence intervals, effect sizes, degrees of freedom and P value noted<br>*Give P values as exact values whenever suitable.*
☐	☒	For Bayesian analysis, information on the choice of priors and Markov chain Monte Carlo settings
☒	☐	For hierarchical and complex designs, identification of the appropriate level for tests and full reporting of outcomes
☒	☐	Estimates of effect sizes (e.g. Cohen's d, Pearson's r), indicating how they were calculated

Our web collection on statistics for biologists contains articles on many of the points above.

Software and code

Policy information about availability of computer code

Data collection	No software was used to collect data in this study.
Data analysis	We used RStudio (4.0.3) and MATLAB (version 2014b). The MATLAB code used for tag data analysis has been released in Cade et al., 2021, Anim Biotelemetry, https://doi.org/10.1186/s40317-021-00256-w. The custom MATLAB code and packages used allows us to calculate whale kinematics and specific scripts help identify the unique kinematic events. The code generated in RStudio will be made available GitHub. The custom R code was used to organize tag data and quantify microplastic ingestion with parameters estimated via Markov Chain Monte Carlo (MCMC) algorithm using MCMCglmm package in R (v 4.0.5, 4.0.5 https://www.r-project.org/).

For manuscripts utilizing custom algorithms or software that are central to the research but not yet described in published literature, software must be made available to editors and reviewers. We strongly encourage code deposition in a community repository (e.g. GitHub). See the Nature Portfolio guidelines for submitting code & software for further information.

Data

Policy information about availability of data

All manuscripts must include a data availability statement. This statement should provide the following information, where applicable:

- Accession codes, unique identifiers, or web links for publicly available datasets
- A description of any restrictions on data availability
- For clinical datasets or third party data, please ensure that the statement adheres to our policy

All data and code will be available on GitHub at https://github.com/shirelkr/risk-of-microplastic-ingestion-by-filter-feeding-megafauna

Field-specific reporting

Please select the one below that is the best fit for your research. If you are not sure, read the appropriate sections before making your selection.

☐ Life sciences ☐ Behavioural & social sciences ☒ Ecological, evolutionary & environmental sciences

For a reference copy of the document with all sections, see nature.com/documents/nr-reporting-summary-flat.pdf

Ecological, evolutionary & environmental sciences study design

All studies must disclose on these points even when the disclosure is negative.

Study description	Our study combined depth-integrated microplastic data from the California Current Ecosystem with high-resolution foraging measurements from 191 tag deployments on blue, fin, and humpback whales in the wild to quantify plastic ingestion rates and routes of exposure. There are no treatment factors or replicates.
Research sample	29 fin whale (Balaenoptera physalus), 126 blue whale (Balaenoptera musculus), and 65 humpback whale (Megaptera novaeangliae) deployments collected between 2010-2019 were used in this study. These tag deployments recorded whales foraging within the California Current Ecosystem, specifically within the Monterey Bay, Channel Islands, Gulf of the Farallones, and Cordell Bank National Marine Sanctuaries. This sample is meant to represent the population of the California Current Ecosystem. The age and gender of these whales is unknown. No manipulation of the organisms occurred. Some of these tag data have been previously published, and can be found here: 1. Cade, D. E. et al. Predator-scale spatial analysis of intra-patch prey distribution reveals the energetic drivers of rorqual whale super-group formation. Funct Ecol 35, 894–908 (2021).<br>2. Goldbogen, J. A. et al. Why whales are big but not bigger: Physiological drivers and ecological limits in the age of ocean giants. Science 366, 1367–1372 (2019).<br>3. Southall, B. L. et al. Behavioral responses of individual blue whales (Balaenoptera musculus) to mid-frequency military sonar. J. Exp. Biol. 222, jeb190637 (2019).<br>4. Calambokidis, J. et al. Differential Vulnerability to Ship Strikes Between Day and Night for Blue, Fin, and Humpback Whales Based on Dive and Movement Data From Medium Duration Archival Tags. Front. Mar. Sci. 6, 543 (2019).
Sampling strategy	Whales were opportunistically tagged and drone images captured opportunistically on their foraging grounds, a total of 191 tagged whales. No sample size calculation was preformed before tagging. At the time of analysis, a sample size calculation in R was performed to determine how many tag deployments were needed for a statistically robust analysis.
Data collection	Data was recorded using CATS and DTAG tags and collected by the paper's authors (Kahane-Rapport, Czapanskiy, Fahlbusch, Friedlaender, Calambokidis, Hazen, Goldbogen and Savoca) . Microplastic data was collected by other authors, papers cited in the Methods section.
Timing and spatial scale	Fieldwork and data analysis was conducted from 2010 to 2019. Tagging occurred when rorqual whales were feeding in the California Current Ecosystem, most often between May to September.
Data exclusions	A tag deployment had to contain feeding events and occur in locations were microplastic sampling data was available to be used.
Reproducibility	All fieldwork, data processing, and analysis methods are given in high-level detail in the manuscript and allow for replication if desired.
Randomization	Randomization was not necessary in this study as this study did not consist of a controlled experiment. This study is an analysis of data collected by wild animals that were not part of an experiment.
Blinding	Blinding was not necessary to this study as this study does not consist of a controlled experiment. The data used was collected by wild animals not part of an experiment.

Did the study involve field work? ☒ Yes ☐ No

Field work, collection and transport

Field conditions	Fieldwork was only conducted on days when the weather permitted small boat operations; only days with minimal to moderate wind and swell, and no rainfall. Fieldwork was conducted in the summer months in California.
Location	Fieldwork (tagging and microplastic collection) was conducted in Monterey Bay, Channel Islands, Gulf of the Farallones, and Cordell Bank National Marine Sanctuaries. Fig. 2 provides both tagging and microplastic sampling locations.
Access & import/export	All fieldwork was conducted under permits issued by the National Marine Fisheries Service (#16111, 14809, 19116, 21678, 20430) and by the National Marine Sanctuary (permit MULTI-2017-007, MULTI-2019-009).
Disturbance	Only when in the process of tagging were whales approached; otherwise a distance of 50m+ was maintained at all times. Drone images were taken at the same time as tagging to minimize disturbance.

Reporting for specific materials, systems and methods

We require information from authors about some types of materials, experimental systems and methods used in many studies. Here, indicate whether each material, system or method listed is relevant to your study. If you are not sure if a list item applies to your research, read the appropriate section before selecting a response.

Materials & experimental systems

n/a	Involved in the study
☒	☐ Antibodies
☒	☐ Eukaryotic cell lines
☒	☐ Palaeontology and archaeology
☐	☒ Animals and other organisms
☒	☐ Human research participants
☒	☐ Clinical data
☒	☐ Dual use research of concern

Methods

n/a	Involved in the study
☒	☐ ChIP-seq
☒	☐ Flow cytometry
☒	☐ MRI-based neuroimaging

Animals and other organisms

Policy information about studies involving animals; ARRIVE guidelines recommended for reporting animal research

Laboratory animals	No laboratory animals were used in this study.
Wild animals	Fieldwork was conducted with fin whales (Balaenoptera physalus), blue whales (Balaenoptera musculus), and humpback whales (Megaptera novaeangliae). Tags were attached via suction cup or dart to foraging rorqual whales in the wild. Drones were used measure whale length. Prey patches were studied using active acoustics from a small boat. All fieldwork was conducted under permits issued by the National Marine Fisheries Service (#16111, 14809, 19116, 21678, 20430) and by the National Marine Sanctuary (permit MULTI-2017-007, MULTI-2019-009).
Field-collected samples	This study did not involve samples collected from the field. This study only analyzed data collected by deployed tags, echosounders, drones, and collected microplastics.
Ethics oversight	Research was conducted in accordance with Stanford University's IACUC (#30123). At Stanford, this committee is known as the Administrative Panel on Laboratory Animal Care (APLAC).

Note that full information on the approval of the study protocol must also be provided in the manuscript.
